# Blockchain Smart Contracts for Automating Clinical Trials: Systematic Review and Proposed System Architecture

**DOI:** 10.2196/76980

**Published:** 2026-05-28

**Authors:** Zara Sheikh, Gargi Samarth, Usman Jaffer

**Affiliations:** 1 Imperial Vascular Unit, St Mary's Hospital, Imperial College Healthcare NHS Trust, Praed Street, London, W2 1NY, United Kingdom, 44 7933513065; 2 Harvard Health Systems Innovation Lab, Harvard University, United States; 3 Anglia Ruskin University, Cambridge, United Kingdom

**Keywords:** blockchain, ethereum, data transparency, smart contracts, clinical trials, drug trials

## Abstract

**Background:**

Blockchain technologies have revolutionized the financial sector through their ability to generate immutable, cryptographically secure records. Clinical trials and health care data possess several synergies with those of the financial sector, specifically pertaining to the importance of tamper-resistant recording of processes. The evolution of blockchain to autonomously execute tasks contingent upon predefined contractual terms via smart contracts (SCs) allows a dynamic chain of interlinked events to unfold independently and in sequence, with time-stamped records. In recent years, mistrust in clinical trial data has grown significantly. Recording the entire clinical trial lifecycle from application, registration, recruitment to conduct, finance management, statistical analysis, and reporting in an immutable, cryptographically secure ledger with SC execution of trial processes could limit the potential for human intervention and tampering. This would produce a time-stamped record of all events within the trial lifecycle. Leveraging the capabilities of SCs could alleviate recruitment challenges and address ongoing concerns regarding data transparency, ownership, and integrity that currently undermine clinical trial processes.

**Objective:**

This study aimed to review the existing literature on SC applications in clinical trials and propose a system architecture for using SCs to automate key processes throughout the clinical trial lifecycle.

**Methods:**

A systematic search was conducted in accordance with the PRISMA (Preferred Reporting Items for Systematic Reviews and Meta-Analyses) guidelines, identifying peer-reviewed studies and open-source repositories pertaining to the implementation of SCs in clinical trials. Data were extracted specific to the stage of the trial lifecycle described, SC architecture, and technical specifications for real-world implementation. Data were synthesized to propose an architecture for automating clinical trial processes within the lifecycle using SCs.

**Results:**

A total of 144 records were screened; 10 studies met the inclusion criteria. Most implementations used private Ethereum-based networks (7/10, 70%). Reported applications included automated patient matching (5/10, 50%), consent management with dynamic permissioning (6/10, 60%), protocol enforcement and time-stamped audit logs (9/10, 90%), adverse event reporting (3/10, 30%), and financial or workflow automation (3/10, 30%). SC-based recruitment systems demonstrated rapid matching performance (eg, 6000 simulated patients matched in 2.13 s). However, all included systems were prototypes or simulations, and none were tested in real-world regulatory settings. Scalability, interoperability limitations, regulatory ambiguity (eg, General Data Protection Regulation right-to-erasure conflicts), and high infrastructural complexity were common gaps noted across studies.

**Conclusions:**

Current evidence suggests that SCs can enhance transparency, traceability, and automation throughout the clinical trial lifecycle. However, the literature remains dominated by simulation-based prototypes, primarily Ethereum-dependent architectures, and lacks analyses of cost-effectiveness, governance, and integration with institutional workflows. Future research should evaluate hybrid architectures, develop interoperability standards, and assess regulatory and ethical implications in real deployments.

## Introduction

The successful execution of clinical trials presents numerous challenges, particularly in patient recruitment, incentivization, and data integrity [1]. Traditional trial infrastructures rely heavily on centralized data management systems that are vulnerable to breaches, human error, and manipulation—undermining the credibility of clinical outcomes [2]. Furthermore, low public awareness, privacy concerns, and fragmented health records impede the identification and recruitment of eligible participants, while nontransparent reimbursement mechanisms and burdensome administrative procedures contribute to poor retention rates [3].

Simultaneously, data transparency remains a core concern. Inconsistent documentation, delayed reporting, and limited traceability can erode trust among regulators, sponsors, investigators, and patients [4]. These operational and ethical concerns highlight the need for secure, automated, and transparent systems to support clinical research.

Blockchain technology offers a compelling solution to these challenges. As a decentralized, cryptographically secure ledger, blockchain enables transparent, tamper-resistant record-keeping across distributed networks. Once data are entered into the blockchain, it becomes immutable, ensuring the authenticity of records such as trial protocols, consent logs, and clinical outcomes [5].

A powerful feature of blockchain systems is the use of smart contracts (SCs), self-executing agreements coded with predefined conditions [6]. In the context of clinical trials, SCs can automate critical processes such as patient matching, consent acquisition, data permissioning, trial monitoring, and stakeholder coordination [7,8]. Their ability to enforce protocol adherence, timestamp events, and track user activity without intermediaries reduces administrative burdens while improving trust, traceability, and auditability.

This review explores the role of blockchain SCs in enhancing transparency, security, and efficiency across the clinical trial lifecycle. Through a systematic evaluation of peer-reviewed publications, preprints, and open-source implementations, we identify key use cases and architectural patterns in SC-based clinical trial systems. Specifically, we examine how SCs have been applied to optimize patient recruitment, streamline consent workflows, improve data sharing and permissions, automate trial governance, and uphold regulatory compliance.

By analyzing a diverse set of implementations, from academic prototypes to open-source repositories, this review highlights both the potential and the limitations of current blockchain applications in clinical research. In doing so, it offers a foundation for future innovations that leverage SCs to build more trustworthy, efficient, and patient-centric clinical trial ecosystems.

## Methods

### Search Strategy

A systematic literature review was conducted in accordance with the PRISMA (Preferred Reporting Items for Systematic Reviews and Meta-Analyses) [9] guidelines to identify peer-reviewed studies and open-source implementations relevant to the use of blockchain SCs in clinical trials. Two independent reviewers (ZS and GS) reviewed all eligible articles across 3 main sources: PubMed, arXiv, and GitHub, yielding 144 articles for screening, as well as manual cross-referencing of reference lists of screened papers. Conflicts were resolved through discussion. Only articles available in the English language were included. Studies were imported into the Covidence systematic review software.

### Eligibility Criteria

Studies were included if they reported on the use of blockchain technology in SCs within any stage of the clinical trial lifecycle. Examples of eligible studies included but were not limited to patient recruitment, consent acquisition, protocol adherence, interinstitutional data sharing, and outcome reporting. Both academic publications and open-source repositories were eligible for inclusion if they contained sufficient technical detail on system architecture, deployment, or simulation. Studies were excluded if they lacked detail on SCs, had inadequate clinical trial relevance, failed to detail implementation methodology, or reported on nonclinical applications.

### Data Extraction

Data were extracted from each included study using a standardized framework to capture study characteristics, blockchain platform, SC structure and function, use of off-chain storage systems, and targeted stage of the clinical trial cycle. Additional technical parameters such as programming language (eg, Solidity), performance simulation outcomes, stakeholder roles, and system testing (where reported) were also recorded. For GitHub repositories, associated documentation, proposals, and code structure were also reviewed.

### Outcomes and Definitions

The primary outcome was the identification and synthesis of architectural and functional use cases for SCs in clinical trial management. These included applications in trial setup, patient matching, consent and permissions, protocol enforcement, data integrity, real-time monitoring, and reporting. Data were synthesized to develop a system architecture for the integration of SCs into the clinical trial lifecycle.

### Study Quality

Academic studies were appraised for methodological rigor based on descriptive completeness, technical transparency, and real-world relevance. For open-source implementations, code completeness, versioning, accompanying documentation, and alignment with described functionality were assessed. Evaluation was performed independently by 2 reviewers (ZS and GS) and cross-checked for consistency. All final inclusions were agreed upon through consensus.

## Results

### Overview

**Figure 1.**
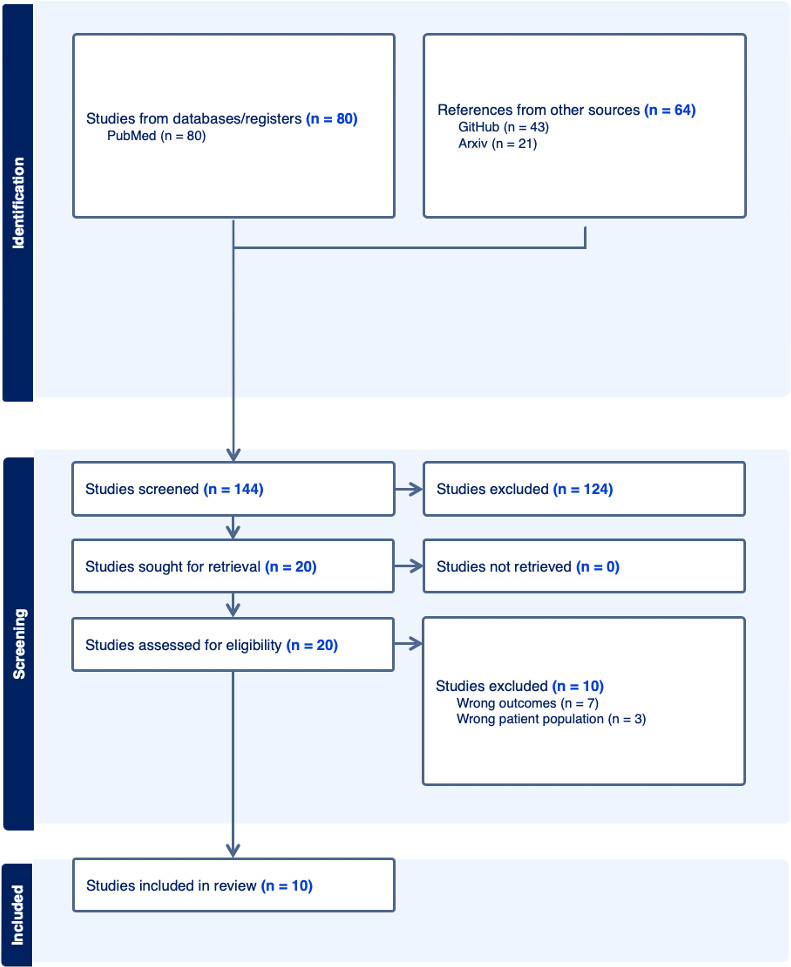
PRISMA (Preferred Items for Systematic Reviews and Meta-Analyses) flow diagram.

In total, 144 records were identified using the search strategy (PubMed=80, GitHub=43, and arXiv=21). Following title and abstract screening, 20 publications (PubMed and arXiv) underwent full-text screening. From this, 6 publications met the inclusion criteria. Following reference screening, a further 2 publications were identified for full-text screening, and one met eligibility criteria. Of the 43 data repositories identified on GitHub, 5 repositories were evaluated after screening, and one was associated with a publication which was included in the final analysis. One further repository detailing the SC code, with an accompanying proposal document, was included in the final analysis. The full search strategy is included in the PRISMA flowchart for study inclusion, shown in Figure 1.

**Figure 2.**
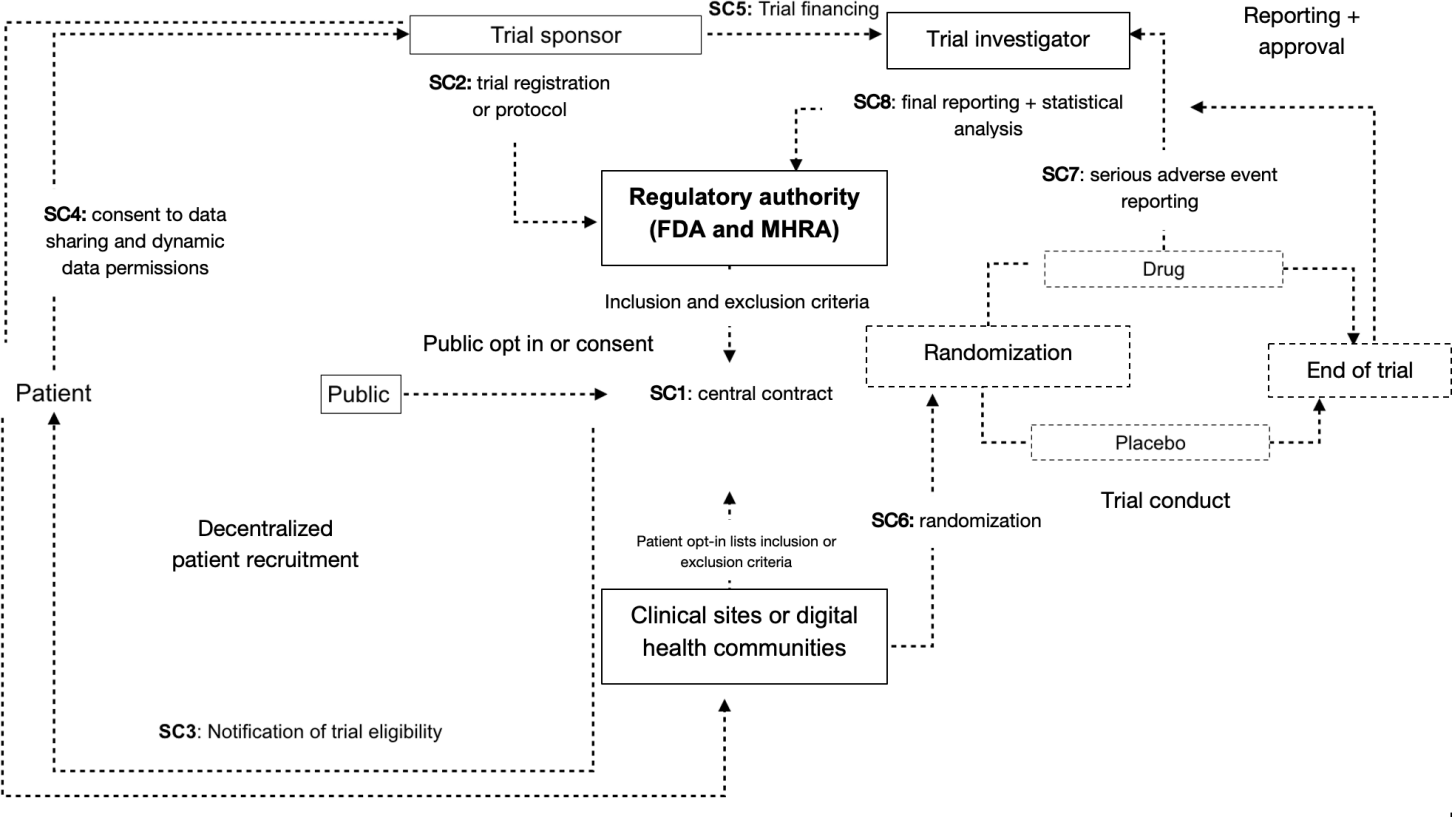
Proposed system architecture for smart contract integration in clinical trials. FDA: US Food and Drug Administration; MHRA: Medicines and Healthcare products Regulatory Agency; SC: smart contract.

Studies detailed several stages of the trial process which could be optimized using SCs. The majority implemented private Ethereum-based blockchains, using Solidity, a programming language specifically designed for writing SCs in Ethereum [10]. One used Quorum, an Ethereum-based blockchain platform, and another used Hyperledger Fabric. Across studies, SCs facilitated trial application [2], patient recruitment [3], consent [4], and dynamic data permissions, allowing patients to grant or revoke access to data [2] (Table 1). SCs were also used to randomize patients [1], set up trial protocols [3], and ensure data transparency [5]. Other applications included trial workflow management [5], reporting of serious adverse events (SAEs) [3], statistical analysis and final reporting [4], data exchange between institutions, and financial management [1]. Several studies [5] described a multilayer architecture with multiple SCs for different stages of clinical trial management (Table 2). Extracted data were synthesized to develop a novel system architecture for the integration of SCs in all aspects of the clinical trial lifecycle (Figure 2).

**Table 1.** Matrix of smart contract features used in clinical trial applications.

Authors (years)		Blockchain	Public or private	Trial application	Trial protocol	Patient recruitment	Randomization double blinding	Consent	Dynamic data permissions	Data transparency and protocol adherence	Trial monitoring	Trial workflow management	Reporting adverse events	Data analysis or final reporting	Data exchange	Financial management
Zhuang et al [11] (2020)		Ethereum	PR^a^	—^b^	—	✓^c^	—	—	—	—	—	—	—	—	—	—
Maslove et al [12] (2018)		Ethereum	PR	—	—	—	—	✓	✓	—	—	—	—	—	—	—
Zhuang et al [11] (2020)		Ethereum	—	—	—	—	—	✓	✓	—	—	—	—	—	✓	—
Zhuang et al [13] (2022)		Quorum Blockchain	PU^d^-PR	—	✓	✓	—	✓	—	✓	✓	✓	—	✓	—	✓
Chaudhry et al [3] (2019)		Hyperledger Fabric	PR	—	—	✓	—	—	—	✓	✓	—	✓	✓	—	—
Nugent et al [8] (2016)		Ethereum	PR	✓	✓	—	—	—	—	✓	✓	—	—	—	—	—
Sendin and Miani [14] (2021)		Ethereum	PR	—	—	—	✓	—	—	✓	✓	✓	✓	✓	—	—
Omar et al [15] (2020)		Ethereum	PR	✓	✓	—	—	✓	—	✓	✓	✓	✓	✓	—	—
Fok [16] (2022)		Polka Dot Substrate	PU	—	✓	—	—	✓	✓	✓	—	—	—	✓	—	—
Hyclem [17] (2019)		Ethereum	PR	—	—	—	—	—	—	—	—	—	—	—	✓	—

a PR: private.

b —: feature not evaluated.

c ✓: feature evaluated.

d PU: public.

**Table 2.** Blockchain applications in clinical trial management: comparative summary of key studies.

Authors (years)	Title	Region	Blockchain	Recruitment	Consent	Trial data management	Features	Technical	Outcomes
Zhuang et al [11] (2020)	Applying blockchain technology to enhance clinical trial recruitment	United States and Taiwan	Ethereum	Smart contract–based patient-trial matching	On-chain E-signature using private key; encrypted identifiers	SCs^a^ manage electronic health record, enrollment, and access control	Master contract for subject matching + individual trial contracts for enrollment and monitoring	Ethereum + remote procedure cell (RPC), EHR^b^ adapters, Solidity or NodeJS or SQL, ABI (application binary interface)-linked GUI^c^, and private chain genesis block	Simulated recruitment (6000 patients); fast, secure, and scalable (~2.13 s match time)
Maslove et al [12] (2018)	Using blockchain technology to manage clinical trials data: a proof-of-concept study	Canada	Ethereum	Web app for permission control (not focused on recruitment)	Dynamic consent via SCs (add or edit permissions)	Patients set data permissions; emphasizes autonomy	Two SCs: patient (permissions) and research (queries); web-based interface	Private Ethereum network, Solidity SCs, web UI^d^, off-chain Oracle	Proof-of-concept; enabled auditability, patient control, and ethics board integration
Zhuang et al [11] (2020)	Generalizable layered blockchain architecture for health care applications: development, case studies, and evaluation	United States	Ethereum	Simulated opt-in matching via SCs	Consent managed via GUI; immutably recorded on-chain	Off-chain EHR with on-chain metadata and data access traceability	3-layer architecture: transaction (SCs), interfacing (metadata exchange), application (trial tools and AI^e^)	Health info exchange via SCs; patient-controlled EHR access; blockchain-based authentication	Simulated recruitment + health information exchange (40,000 patients, 331K transactions); avg. 3.07s consent
Zhuang et al [13] (2022)	Re-engineering a clinical trial management system using blockchain technology: system design, development, and case studies	China, United States, and Taiwan	Quorum + IPFS^f^	Automated matching via SCs + digital consent	Digital signature verified and stored on-chain	End-to-end trial system; data in IPFS, hashes on-chain	Clinical trial management systems with SCs for 5 trial phases: planning, recruitment, conduct, reporting and finance	Quorum + Raft, SCs, IPFS, blockchain GUI, 6-node sponsor or testbed setup	21.6M transactions, 100% success, 335.4 TPS; scalable and secure in real-world scenarios
Chauhdhry et al [3] (2019)	A blockchain framework for managing and monitoring data in multi-site clinical trials	United States	Hyperledger Fabric	Eligibility-based enrollment via subject channel	Private channel stores PHI^g^; access tied to deidentified info	Emphasis on data security and cost optimization	Audit trail, privacy, protocol enforcement, and adverse event reporting	Hyperledger Fabric (orderer or peer or client nodes), Practical Byzantine Fault Tolerance (PBFT) or proof-of-elapsed-time (PoET) consensus, private ledgers, auto chaincode	Meets NIH^h^ guidelines; supports versioning, privacy, audit trails, adverse event tracking
Nugent et al [8] (2016)	Improving data transparency in clinical trials using blockchain SCs	United Kingdom	Ethereum, IPFS, Swarm	SCs restrict recruitment to approved period	Encrypted documents on-chain; future use of zero knowledge proofs proposed	Protocol integrity and subject tracking via SCs	Immutable logs, tamper-proof protocol, auto-deploy contracts, subject tracking	Private Ethereum deployment for Tamiflu trial simulation, append-only logs, 14-s block time, historic query support	Private Ethereum trial logs; verifiable protocol adherence and timestamped data on number of participants and trials at any time
Omar et al [15] (2020)	Ensuring protocol compliance and data transparency in clinical trials using blockchain SCs	United Arab Emirates	Ethereum, IPFS	Physician-controlled enrollment via verified addresses	Hashed consent via IPFS; traceable by Ethereum address	Staged trial process with IPFS-based immutable records	Decentralized control: SCs, IPFS, access via Ethereum addresses	Remix integrated development environment (IDE), solidity, open-source modular trial framework (eg, crossover, placebo)	Validated system with high compliance, traceability, and role-specific access
Sendin and Miani [14] (2021)	Toward reliable and transparent vaccine phase III trials with SCs	Brazil	Ethereum	Randomized matching using commitment schemes	Implicit via registration; confirms status without disclosure	Fully on-chain illness registration and shot tracking	Double-blind randomization, fraud prevention, public auditability	Solidity+ Brownie+ Python frontend, open-source VaccSC repo	Transparent, tamper-proof Phase III vaccine trial protocol; publicor private use-ready
Fok [16] (2022)	Clinical trial data smart contract	United States and Europe	Substrate, Polka dot	Enrollment via smart contract forms linked to wallets	Dynamic consent via smart contract; supports revocable access	Off-chain data with on-chain hashes and consent metadata	Wallet-linked form submission, verifiable logs, modular design with Rust + React	Rust SCs (Substrate), React frontend, Polkadot.js integration, optional IPFS	Full-stack decentralized trial system with modern Web3 stack
Hyclem (2019) [17]	EssaisCliniques smart contract for blockchain-based clinical trial registration and feedback	Europe	Ethereum	Physician enrollment after registration	Consent implied via physician action; no cryptographic proof	On-chain patient-drug mapping and feedback via smart contract	Role-based access, drug-patient mapping, feedback submission via smart contract	Solidity (v0.4‐0.6), access via mappings, trial data in struct arrays, no encryption or access control	Basic proof-of-concept; enforces roles and feedback, lacks advanced security

a SC: smart contract.

b EHR: electronic health record.

c GUI: graphical user interface.

d UI: user interface.

e AI: artificial intelligence.

f IPFS: interplanetary file system.

g PHI: protected health information.

h NIH: National Institutes of Health.

### Patient Recruitment

Recruitment for clinical trials is frequently challenging, often constrained by bureaucratic processes, multisite coordination requirements, and reliance on site-based recruiters. These inefficiencies hinder enrollment, delay trial timelines, and raise study costs. Patient enrollment is also hindered by a lack of awareness regarding available trials, exacerbated by inadequate outreach and poor trial visibility. Moreover, the necessity of additional hospital visits to discuss trial protocols can act as a further deterrent to participation.

SCs can be used to “auto-match” eligible patients to suitable clinical trials, thereby streamlining the recruitment process. Zhuang et al [7] propose a 2-tier blockchain architecture consisting of a Master Smart Contract and multiple Trial Contracts. The Master Smart Contract is managed by a trusted authority, such as the US Food and Drug Administration (FDA), and is designed to match patients to clinical trials based on predefined inclusion and exclusion criteria. To ensure trial authenticity and regulatory compliance, each sponsor is required to submit a transaction to the authority. This transaction includes a National Clinical Trial identifier and supporting trial information for validation. Once the authority has verified the sponsor’s identity and authenticity of the trial, it can execute the master smart contract, based on inclusion or exclusion criteria submitted by the sponsor. Patient information is inputted by clinical sites for patients who have opted in for consideration of trial participation. This enables the system to automatically match eligible patients to appropriate trials.

Once a patient is matched, they are notified by the authority that there is a clinical trial they may be eligible to participate in. Patient authorization is subsequently required to permit sponsor access to electronic health records (EHRs). Sponsors can then verify that the patient information supplied to the authority matches their clinical records and precise matching can be performed by the clinical site. If patients fully match, the sponsor can send the patient a transaction to enroll in the trial which can be authenticated using an eSignature. Patient records are then stored in the trial contract. Trial-specific contracts also contain information on trial protocols, sponsor details, and other relevant documentation. Importantly, trial contracts are only accessible to individuals who have agreed to participate in the trial.

The authors simulate the use of the master contract, demonstrating the ability to auto-match 1145 of 6000 patients to a trial in 2.13 seconds. Once patients have enrolled using a trial-specific contract, the sponsor can access data on the geographical location of patients and choose clinical trial sites convenient to the majority of subjects. This significantly attenuates the time and resources associated with patient screening processes, widening accessibility and trial awareness.

### Data Permissions and Consent

Lack of transparency on how health care data are used can lead to mistrust in the clinical trial process. Blocktrial, a web-based application developed by Maslove et al [12], is a simple patient SC that enables patients to specify data sharing permissions. These permissions can be changed through an “editPermissions” feature on the patient SC, allowing patients to control who has access to their data. Once consent to data sharing is edited, an immutable record is generated on the blockchain. SCs allowing real-time editing of data sharing permissions promote patient autonomy and agency, and may enhance trust and transparency in how patient data are used.

### Protocol Compliance and Data Transparency

While patient-centric models offer valuable enhancements to consent management, broader systemic trust requires secure, verifiable collaboration between institutions involved in clinical trials. To this end, Nugent et al [8] propose a private, permissioned Ethereum-based blockchain network jointly maintained by regulatory bodies (eg, Medicines and Healthcare products Regulatory Agency and FDA), pharmaceutical companies, and contract research organizations (CROs) [8]. This network is intended to operate alongside traditional clinical data management systems, serving as a transactional, interorganizational record-keeping system. The authors illustrate that SCs can function as trusted administrative agents, enhancing transparency in clinical trial reporting by immutably recording key elements prone to manipulation, such as trial registration, protocols, subject enrollment, and clinical measurements. Within this architecture, Nugent et al [8] outline a hierarchical SC model comprising two core types:

1. Regulator contract: this SC maintains a data structure that holds Clinical Trial Authorization details. It is controlled and updated by regulatory authorities such as the Medicines and Healthcare products Regulatory Agency or the FDA, based on off-chain licensing agreements. The contract also includes a container to store individual trial contracts.
2. Trial contract: this is executed by the CROs through a function in the regulator contract; this contract’s deployment is governed by permissioning logic tied to the Clinical Trial Authorization data. It holds a data structure for storing the trial protocol, leveraging the interplanetary file system (IPFS) or Ethereum’s Swarm protocol for large file storage. The contract enforces rules requiring the protocol to be deposited and trial endpoints defined before subject data can be added to the associated container.

Finally, a trial report is generated, reading all data from the blockchain, with details of each subject and data points that have been added with timestamping. Such systems can strengthen trust in clinical data integrity and improve the credibility of trial outcomes, ultimately enabling more informed medical decisions while helping to reduce patient risk and the financial burden caused by data manipulation.

By leveraging the cryptographic security of modern blockchain protocols, the system extends beyond simple proof-of-existence to support complex, tamper-resistant data handling. This work illustrates how SCs on the Ethereum blockchain can enhance transparency in clinical trial data management.

As part of their evaluation, Nugent et al [8] deployed SCs on a private Ethereum blockchain to record synthetic data simulating clinical trials for Tamiflu, a medication stockpiled by the British government at a cost of US $575 million, despite 60% of the trial data being unpublished at the time. This test demonstrated the system’s ability to provide real-time access to trial data, including participant numbers, number of trials, transaction addresses (linked to a CRO), and timestamps, while the blockchain’s immutability allowed for retrieval of historical data, confirming the viability of using SCs to maintain transparent, verifiable records in clinical trials.

Similarly, Omar et al [15] propose a blockchain-based clinical trial framework using Ethereum SCs and IPFS to enhance transparency, traceability, and automation in trial data management. IPFS is used to store trial documents, such as protocols, consent, SAE reports, and lab results, while their cryptographic hashes are securely linked to the blockchain via SCs. These SCs automate critical tasks at each stage of the trial process, such as Investigational New Drug submission, trial initiation, patient enrollment, monitoring, and SAE occurrence. At the end of the trial, the sponsor is required to submit an analysis to the SC, following which the FDA can approve the final outcome.

Each stakeholder (eg, FDA, institutional review board, sponsor, and physician) is assigned a unique Ethereum address, enabling secure, permissioned interactions with the contract’s specific functions, such as submitting data or accessing trial updates. The contracts enforce role-based access controls, ensuring that only authorized individuals can perform actions within the system. Events, such as patient enrollment or trial stage completion, trigger real-time notifications to the relevant stakeholders, enhancing communication and transparency throughout the trial lifecycle. By automating these processes, SCs reduce administrative bottlenecks, improve data integrity, and ensure the auditability of every action taken in the clinical trial.

The authors successfully tested this SC framework across various scenarios in the clinical trial process. At each stage, from Investigational New Drug submission to final analysis, the system correctly executed role-specific tasks with access controls in place to prevent unauthorized actions. If an unauthorized action was attempted, the SC triggered an error, ensuring that only valid operations could be performed. Additionally, real-time notifications were generated for stakeholders at key points, such as patient enrollment or SAE reporting. The framework also enabled the retrieval of patient monitoring data per visit and correctly managed patient dropouts by preventing further data updates for removed participants. SAEs were reported by the principal investigator, reviewed by the institutional review board, and the final decisions were broadcast to all stakeholders through blockchain events. The simulated trial concludes with the FDA issuing a decision based on the final report, triggering an event that informs all stakeholders of the outcome, further demonstrating the ability of Ethereum SCs to manage clinical trials in a secure, transparent, and auditable manner.

### SCs in Vaccine Development

Mistrust in vaccine development, driven by concerns over data transparency and trial integrity, remains a major challenge to public confidence. Sendin et al [14] attempt to address this through vaccine supply chain (VaccSC), using SCs to securely automate and coordinate key stages of a phase III vaccine trial while preserving double-blind conditions.

VaccSC leverages SCs on the Ethereum blockchain to automate randomization and requires the developer to specify the number of participants, the infected threshold (number of patients infected before allocations are revealed), minimum vaccine efficacy for approval, and the Ethereum address of clinics administering the vaccine in the SC before deployment. Once deployed by the vaccine developer, the SC governs the entire trial workflow. First, the developer distributes vaccine and placebo shots to clinics and records their association on-chain. Randomization is achieved through private number generation by the patient and the clinic; both numbers are then committed to the SC and combined to give a unique number, assigning a shot to the patient. Clinics then administer the shots and link unique shot identifiers to each patient within the contract, which the patient confirms. If a patient becomes ill, they report this directly to the contract. When a predefined infection threshold is met, the SC emits an event notifying the vaccine developer that the trial is complete. Using Ethereum addresses, the developer submits which patients received the control, allowing the contract to calculate how many received the actual vaccine. The contract then computes vaccine efficacy and approval status based on previously defined thresholds, making the results publicly accessible. By immutably capturing each step, from shot distribution, randomization, infection rates, and efficacy calculations, VaccSC uses SCs not only as a secure data ledger but also as an autonomous, unbiased administrator, reducing the risk of data manipulation and making the process transparent and accessible to the public. This methodology can be replicated in other drug development settings.

### Multilayered SC Architectures for Comprehensive Trial Oversight

Several studies describe a multilayered approach to clinical trial management. Zhuang et al [11] initially proposed a 3-level architecture, consisting of a transaction, interface, and application layer. The transaction layer consists of 2 SCs, the EHR manager and the user manager. The EHR system automatically extracts patient ID, hospital ID, patient name, and a dataset related to patient health care use as an execution function of the SC on receiving a new EHR from a health institution. This data are then submitted to the blockchain. The user manager enables patients to grant institutions access to their metadata. Once access permission is verified, the blockchain adaptor will automatically retrieve the patient’s metadata from the EHR manager, request the encrypted EHR dataset from the remote health facility in which it is stored, retrieve the encrypted data using dataset location information from the EHR metadata, and decrypt the data.

The interfacing layer enables secure data exchange between institutions through 4 functions. These include accessing external data, storing encrypted data off-chain, posting metadata and access requests to the blockchain via SCs, and transmitting encrypted data to authorized recipients. It retrieves metadata from the blockchain, verifies data integrity, and decrypts off-chain data as needed. The data retrieval and permission setting application programming interface calls on the SCs in the transaction layer to retrieve metadata from the blockchain and set access policies.

The application layer relies on the transaction SCs and interfacing layer to securely access data applications, which can be developed to interact with the preceding layers, such as data analytic tools to match patients to relevant trials or for patients to grant or revoke access to their records and track how they are being used.

Building on their earlier 3-layer architecture, the authors outline a more granular SC-based framework spanning five phases of the clinical trial process: (1) study planning, using a transparent electronic trial master file for protocol development; (2) study start-up, including patient recruitment and enrollment; (3) study conduct, supporting electronic data capture and monitoring of treatment safety and efficacy; (4) study closeout, incorporating statistical tools for reproducible analytics reporting; and (5) study finance, which leverages blockchain’s cryptocurrency capabilities for payment and reimbursement [13].

The study planning stage uses SCs to control access to electronic documents relevant to the trial, validate file consistency, and manage collaboration in electronic trial master file development, and the IPFS network is used for file storage and file indexing. Trial master file documents are identified in the SC at the start of the trial, and sponsors are required to add the blockchain account of users who are permitted to work on the file. The SC only permits 1 user at a time to work on a document, ensuring traceability and auditability of any modifications to trial documents. Once a user has finished working on a document, the blockchain adaptor will encrypt the document with a random new set of keys.

The study start-up phase builds on earlier work using SCs to automate matching and enhance recruitment. During the conduct phase, patient data are entered on an electronic case report form following each clinic visit; these are automatically hashed and stored in the IPFS. The SC determines whether a trial site has permission to store a patient’s data. Once verified, the SC will send a visit ID and decryption key through the Quorum blockchain’s private transaction. The sponsor will receive the decryption key and hash from the blockchain, and the blockchain adaptor will automatically decrypt and hash the records to compare with the hash stored on the blockchain, thereby identifying hash mismatches and data inconsistencies. Mismatches trigger alerts for investigation. The blockchain’s immutability prevents overwriting erroneous data; instead, linking it to previous record hashes for validation.

The closeout phase involves encoding statistical analysis methods into an SC, with both the analyzed data and methods stored on the blockchain for reproducibility and result authentication. The final component pertains to finances, encoding a list of payable entities and predefined rates into an SC at the start of the study. This standardization reduces the risk of hidden fees during the trial and can be customized to the trial site to accommodate different rates. Encoding payables into the SCs can expedite the validation process. Furthermore, all financial transactions are recorded and time-stamped on the blockchain, allowing transparency around trial finances.

## Discussion

### Principal Findings

In recent years, public confidence in the validity of clinical research has been challenged, with multiple reports documenting that a substantial proportion of published studies contain methodological flaws or, in some cases, fabricated data [2]. This erosion of trust has intensified interest in technologies capable of enhancing transparency, auditability, and accountability across the clinical trial lifecycle. Since Satoshi Nakamoto’s [18] 2008 white paper introduced a decentralized, time-stamped ledger for peer-to-peer financial exchange, blockchain technology has transformed the financial sector and has increasingly been explored for its potential applications in health care data management [19]. The fundamental properties of decentralized data storage, cryptographic integrity, and tamper-resistant audit trails have drawn attention as possible mechanisms for strengthening trust in clinical trials.

The emergence of SCs extends these capabilities from static, “proof-of-existence” records to programmable logic, capable of automating key operational processes. SCs can function as autonomous administrators of trial workflows, implementing predefined rules, and eliminating opportunities for retrospective alteration. This embodies Lawrence Lessig’s idea that “code is law,” such that the software logic itself becomes the governing framework for how stakeholders interact. Within the context of clinical trials, this has motivated the development of prototype systems that automate tasks ranging from eligibility screening and recruitment to dynamic consent management, protocol enforcement, data access tracking, and even automated reporting of results.

The studies included in this review demonstrate that many such applications are technically feasible in controlled environments. For example, SC-based recruitment workflows have been shown to match large numbers of simulated participants to trial criteria within seconds, and immutable audit trails can capture the entire sequence of procedural steps across the trial lifecycle. Dynamic data permissioning enabled by SCs may strengthen patient autonomy by allowing individuals to regulate access to their data in real time. The Ethereum blockchain, used in the majority of reviewed studies, has proven well-suited to these prototypes due to its mature SC infrastructure and developer ecosystem.

However, the findings also reveal a substantial gap between technical promise and real-world readiness. All identified implementations were prototypes or simulations, with no evidence of deployment in actual regulated trials. Assertions that clinical trial processes could be fully automated should therefore be interpreted with caution. Although the reviewed systems demonstrate the potential of SCs to automate constrained, deterministic processes, they do not address the complexities of real-world trial environments, such as the need for human judgment, protocol deviations, unstructured clinical data, and the heterogeneity of institutional workflows. Furthermore, Ethereum’s dominance in the literature introduces platform bias, and few studies critically examine its well-known limitations, including fluctuating transaction costs, constraints on throughput, and the absence of built-in privacy protections.

Regulatory and legal challenges present even greater obstacles. The immutability of blockchain data conflicts with requirements under frameworks such as the General Data Protection Regulation, which grant individuals the right to erasure or correction of their personal data. While hybrid on-chain or off-chain architectures offer partial solutions, they also reintroduce trust dependencies that undermine decentralization. In addition, governments may impose abrupt regulatory changes or restrictions on blockchain platforms, as demonstrated by cryptocurrency bans in China and other jurisdictions. Such policy shifts pose risks to any clinical trial infrastructure built on blockchain technology. Adoption will therefore require not only technical adaptation but also careful consideration of governance, compliance, and stakeholder engagement.

Another critical gap in the current literature is the lack of evidence regarding stakeholder perspectives. No included study evaluated how patients, investigators, regulators, data monitors, or ethics committees perceive blockchain-based systems. The introduction of SCs could alter roles, responsibilities, and liabilities within clinical trials, and resistance to such changes may limit adoption. Moreover, the reviewed studies did not assess cost-effectiveness, integration with existing EHR or clinical trial management systems, or long-term sustainability, all of which are essential for real-world implementation.

Taken together, the findings indicate that blockchain SCs offer meaningful conceptual advantages, particularly in transparency, auditability, and automation, but their practical value remains unproven. Future progress will require a shift from idealized prototypes to rigorous evaluation within real clinical and regulatory environments, informed by legal expertise, operational realities, and stakeholder acceptance. The transition to blockchain-enabled clinical trials, if it occurs, will not be a purely technical transformation but a broader organizational and regulatory evolution involving new models of data governance and oversight.

### Limitations

This review is subject to several limitations. First, the number of eligible studies was small, and all relied on prototype or simulation-based evaluations, which limits the generalizability of the findings. Second, given the technical nature of the field, important developments may exist outside traditional academic publishing channels. Although GitHub repositories were included, some relevant implementations may not have met the inclusion criteria due to insufficient documentation. Third, the heterogeneity of study designs, blockchain platforms, and evaluation methods precluded quantitative synthesis and necessitated a qualitative approach. Fourth, because the field is evolving rapidly, newer implementations may have emerged after the final search date. Fifth, no study included direct engagement with regulators, trial sponsors, investigators, or patient groups, meaning the review could not assess acceptability or practical feasibility from a stakeholder perspective. These limitations reflect the early stage of this research area and reinforce the need for more comprehensive, empirically grounded studies.

### Future Directions

Future research should prioritize real-world pilot implementations that integrate SCs within existing clinical trial infrastructures, including EHRs, electronic data capture systems, and regulatory submission platforms. Development of privacy-preserving blockchain technologies, such as zero-knowledge proofs, secure multiparty computation, or trusted execution environments, will be essential for reconciling immutability with data protection requirements. Comparative studies evaluating different blockchain platforms, including permissioned and permissionless architectures, would help determine appropriate use cases and clarify the trade-offs between privacy, performance, and decentralization.

Hybrid systems that combine blockchain components with traditional clinical trial management frameworks offer a promising compromise and warrant further exploration. Researchers should also examine governance models for blockchain-enabled trials, ensuring that the encoded logic reflects clinical, ethical, and regulatory expectations. Economic evaluations will be essential to determine whether SC systems can deliver cost savings or improved efficiency relative to conventional solutions. Finally, engagement with stakeholders, including regulators, ethics committees, clinicians, patients, and data managers, will be vital to understand practical adoption barriers and align technical development with real-world priorities.

### Conclusions

Blockchain-based SCs have the potential to revolutionize the way clinical trials are operated, audited, and disseminated. Through unrealized resources and finance efficiencies, recruitment to trials, trial timelines, and conduct could be optimized. The immutable core of the blockchain ledger, greater data decentralization, and patient data ownership could lead to greater trust and transparency in the body of scientific work produced. This review demonstrates that nearly all elements of a clinical trial could be efficiently executed using SC and blockchain technologies.

## Supplementary material

10.2196/76980 Checklist 1 PRISMA checklist.

## Abbreviations

CRO: contract research organization
EHR: electronic health record
FDA: US Food and Drug Administration
IPFS: interplanetary file system
PRISMA: Preferred Reporting Items for Systematic Reviews and Meta-Analyses
SAE: serious adverse event
SC: smart contract
